# Supplementary material for: Single-cell RNAseq identifies clonally expanded antigen-specific T-cells following intradermal injection of gold nanoparticles loaded with diabetes autoantigen in humans
Source: Front Immunol. 2023 Oct 16;14:1276255. doi: 10.3389/fimmu.2023.1276255 (PMC10613693; doi:10.3389/fimmu.2023.1276255)
Supplement: Supplementary file 1 [file DataSheet_1.pdf]

Supplementary Figures

1 Supplementary Figure 1

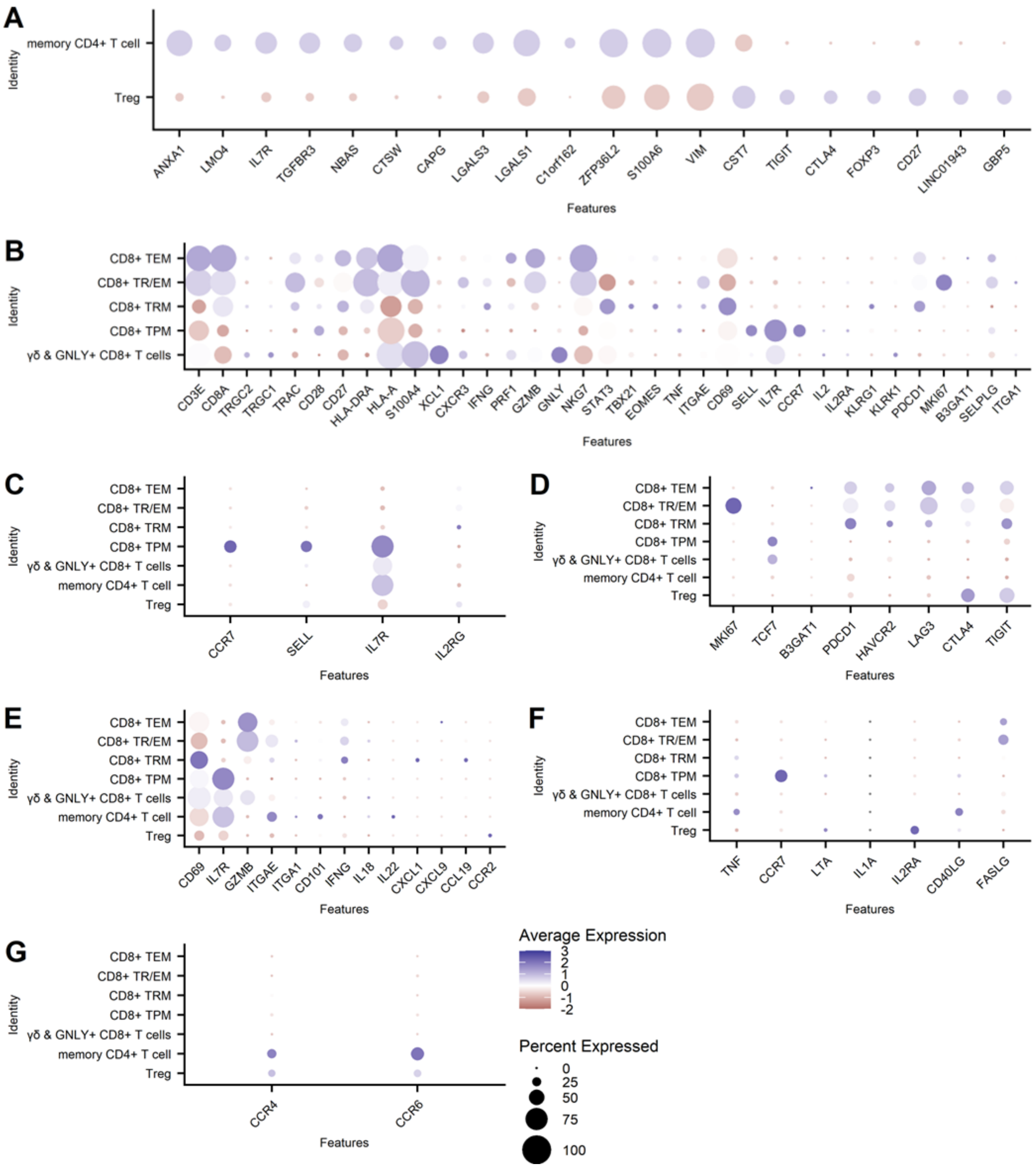

Supplementary Figure 1. Phenotypes of cell subsets presented in dot plots: A)CD4$^+$ cells were manually gated into two subsets and their identities were determined to be Tregs and memory CD4$^+$ T-cells based on the top 20 DEG between the two subsets; B) Key markers of CD8$^+$ T-cell subset identified based on canonical markers expression of memory, resident memory, peripheral memory and effector subsets and those found in the skin(43)**;** C) naïve cell markers; D) exhaustion and proliferation markers in the T-cells; E) expression of genes known to be highly expressed in pancreatic resident T-cells; F) expression of genes known to have low expression in pancreatic resident T-cells; G) expression of skin homing chemokine receptors.

2 Supplementary Figure 2

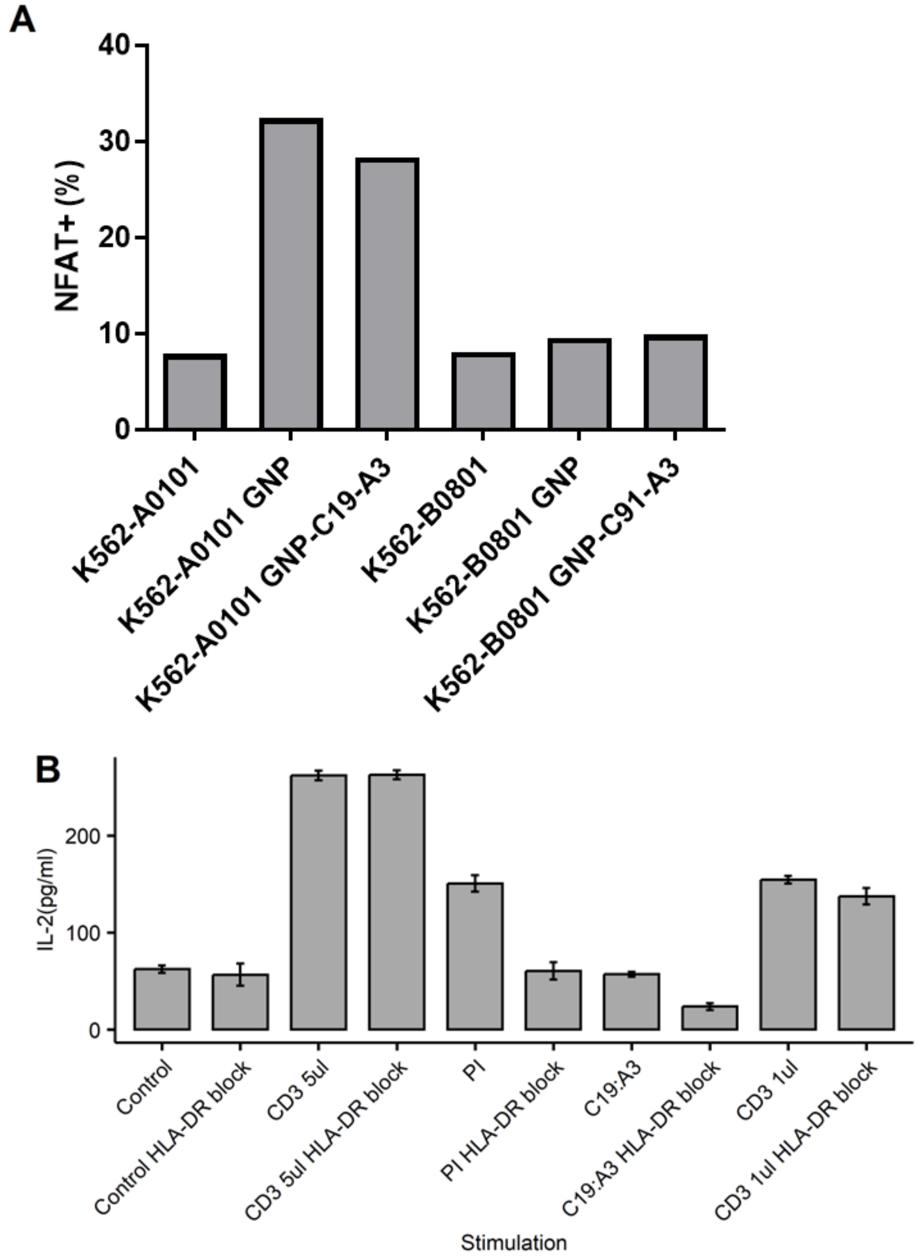

Supplementary Figure 2: Example HLA restriction in GNP and PI- reactive TCRs A) Clone EEASI-A:Bl3 is HLA A01:01 restricted. 5KC expressing the EEASI-A:Bl3 TCR were cultured with K562 cells that expressed either A01:01 or B08:01 (non-relevant HLA) either alone or with GNP or GNPC19-A3. Percentage fluorescence of the NFAT reporter was assessed after 24 hours by flow cytometry. B) Clone EEASI-C:Bl6 is HLA-DR restricted: Control - 5KC plus patient-autologous EBV transformed B cells; Control HLA-DR block – anti-HLA-DR blocking antibody present; CD3 5µL: 5 µL αCD3/CD28 microbeads present as positive control; PI:proinsulin 50µg/ml; C19:A3 – CD19-A3 peptide at 50µg/ml; CD3 1µL: 1µL αCD3/CD28 microbeads submaximal stimulation (not affected by HLA-DR block). HLA-DR block - Ultra-LEAF Purified anti-human HLA-DR Antibody at final concentration of 1.25µg/ml. Results are from single experiments representative of three independent experiments.

3 Supplementary Figure 3

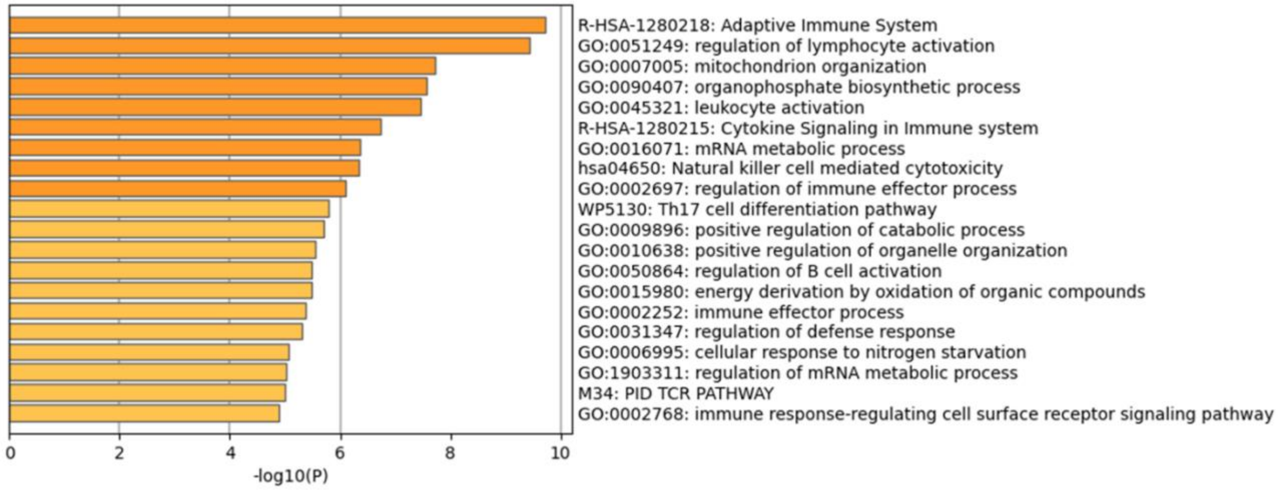

Supplementary Figure 3. DEG between PI and GNP/non-specific T-cells in scRNAseq of blisters. DEG were identified using Seurat and all DEG with an uncorrected p value <0.05 were mapped to pathways in Metascape. The top 20 pathways are shown.

4 Supplementary Figure 4

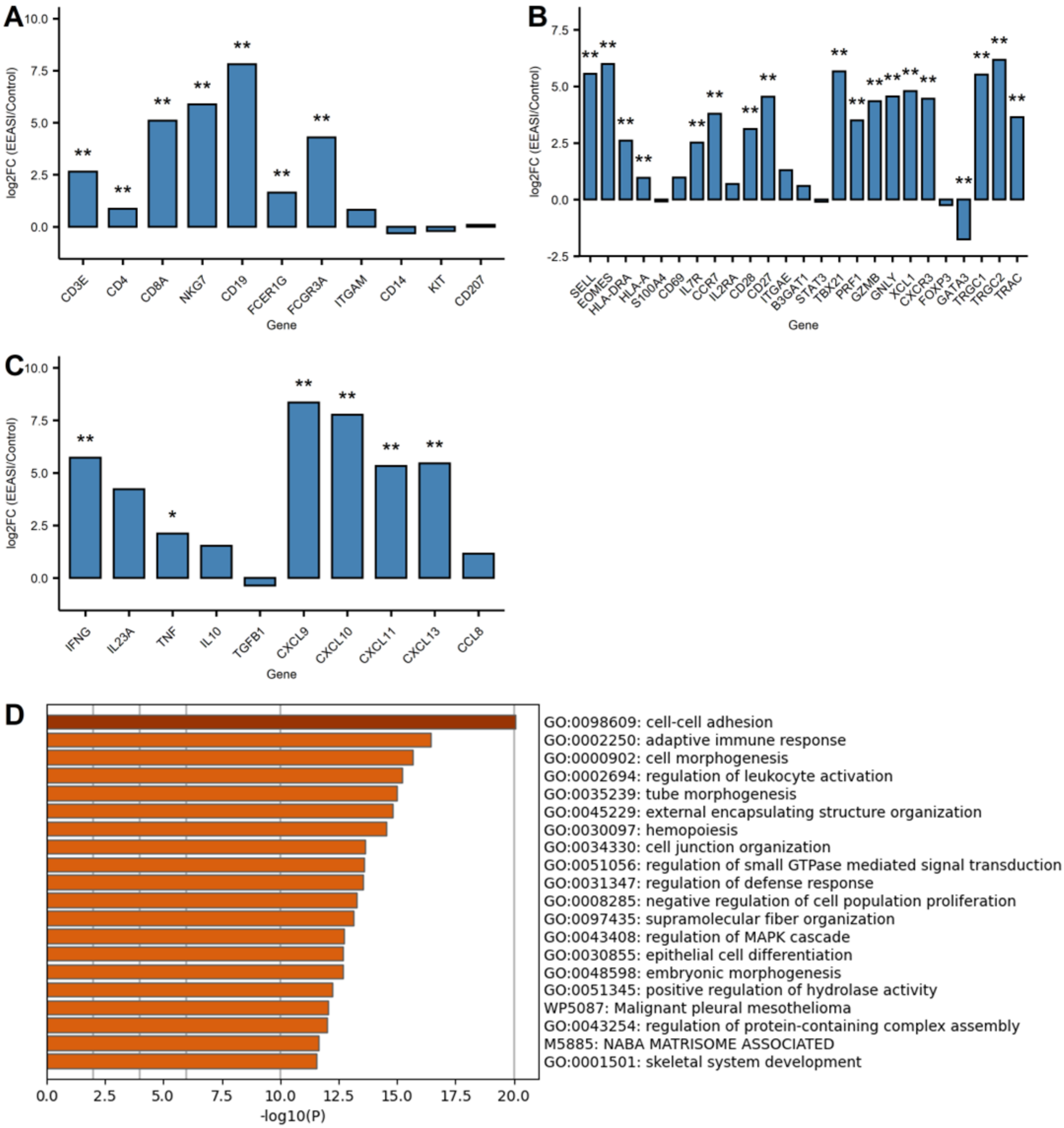

Supplementary Figure 4. Immune cell and skin cell transcripts are differentially expressed between study participants and controls. Bulk RNAseq analysis was performed on punch biopsies from the injection site of three experimental study participants. Mastectomy skin samples, surplus to those required for diagnostic pathology, were used as controls. RNAseq analysis was performed to

sequence all RNA transcripts and the log2 fold change (study participants/controls) in A) Leukocyte markers B) T-cell subset markers C) chemokines and cytokines. *p<0.05 **p<0.01 unpaired T test, FDR corrected d) All differentially expressed genes at p<0.05 FDR corrected, were mapped using Metascape analysis. The top 20 pathways are shown on the graph.

5 Supplementary Figure 5

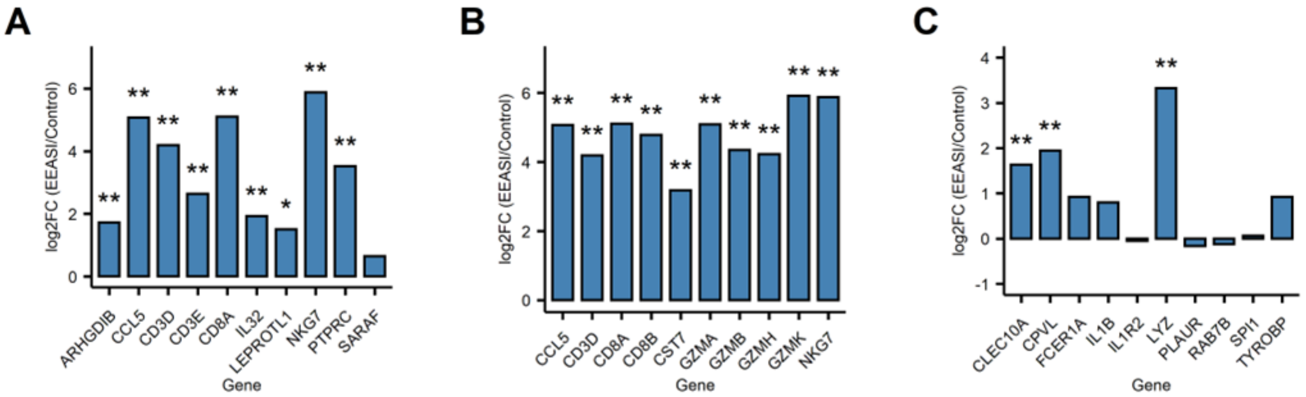

Supplementary Figure 5. Immune cell and skin cell transcripts are differentially expressed between study participant and control based on marker genes from the scRNA seq. The top 10 DEG that defined A) all T-cells, B) CD8+ T_{EM} and; C) DC were identified in the scRNAseq dataset. The relative expression of these genes between punch biopsy RNAseq between experimental study participants and control participants was then determined. *p<0.05, **p<0.01 unpaired T Test, FDR corrected.
