# Supplementary material for: Single-cell RNAseq identifies clonally expanded antigen-specific T-cells following intradermal injection of gold nanoparticles loaded with diabetes autoantigen in humans
Source: Front Immunol. 2023 Oct 16;14:1276255. doi: 10.3389/fimmu.2023.1276255 (PMC10613693; doi:10.3389/fimmu.2023.1276255)
Supplement: Supplementary file 2 [file Table_1.docx]

| Participant Study ID | Age at enrolment in the study (years) | Age at diagnosis (years) | Duration of diabetes at study enrolment (months) | HbA1c at study enrolment  (mmol/mol) | Number of injections | Time between injection and punch biopsy  (months) | Time between injection and suction blister  (months) |
| --- | --- | --- | --- | --- | --- | --- | --- |
| EEASI-A | 27 | 27 | 10 | 40 | 3 | 7 (*1^st^) | 28 (*2^nd^) |
| EEASI-B | 20 | 19 | 7 | 66 | 3 | 3 (*1^st^) | 10 (*2^nd^) |
| EEASI-C | 36 | 33 | 45 | 64 | 1 |  | 10 (*2^nd^) |

**Supplementary Table 1. Demographic and clinical characteristics of participants with timing of study procedures.** *number of injection after which biopsy/suction blister was taken
