# Supplementary material for: Single-cell RNAseq identifies clonally expanded antigen-specific T-cells following intradermal injection of gold nanoparticles loaded with diabetes autoantigen in humans
Source: Front Immunol. 2023 Oct 16;14:1276255. doi: 10.3389/fimmu.2023.1276255 (PMC10613693; doi:10.3389/fimmu.2023.1276255)
Supplement: Supplementary file 3 [file Table_2.docx]

| EEASI information |  |  |  |  |  |  |  |  |  | Database Information |  |  |  |  |  |  |
| --- | --- | --- | --- | --- | --- | --- | --- | --- | --- | --- | --- | --- | --- | --- | --- | --- |
| Donor ID | Clonal expansion | Proportion | Matched cdr3 | TCR ID | T cell origin | Specificity | TCRalpha | TCRbeta | Clonotype ID | Source | CDR3.alpha.aa | CDR3.beta.aa | Pathology | Antigen protein | Epitope peptide | MHC |
| EEASI-A | 7 | 0.025830258 | CAVKNYGQNFVF | EEASI-A:Bl3 | CD8 | GNP | CAVKNYGQNFVF | CSVVTHYGYTF | clonotype3 | VDJDB | CAVKNYGQNFVF | NA | EBV | EBNA4 | AVFDRKSDAK | HLA-A*11:01 |
| EEASI-A | 6 | 0.022140221 | CAVDTGRRALTF | EEASI-A:Bl5 | CD8 | GNP | CAVDTGRRALTF | CAIPLAMNTEAFF | clonotype5 | VDJDB | CAVDTGRRALTF | NA | HomoSapiens | MLANA | ELAGIGILTV | HLA-A*02:01 |
| EEASI-A | 6 | 0.022140221 | CAVDTGRRALTF | EEASI-A:Bl5 | CD8 | GNP | CAVDTGRRALTF | CAIPLAMNTEAFF | clonotype5 | VDJDB | CAVDTGRRALTF | NA | CMV | pp65 | NLVPMVATV | HLA-A*02 |
| EEASI-A | 2 | 0.007380074 | CASSLTSGNEQFF | EEASI-A:E4 | CD4 | PI | CALNTPYSGAGSYQLTF | CASSLTSGNEQFF | clonotype15 | VDJDB | NA | CASSLTSGNEQFF | InfluenzaA | M | GILGFVFTL | HLA-A*02:01 |
| EEASI-A | 2 | 0.007380074 | CASSLTSGNEQFF | EEASI-A:E4 | CD4 | PI | CALNTPYSGAGSYQLTF | CASSLTSGNEQFF | clonotype15 | McPas | NA | CASSLTSGNEQFF | Influenza | D009980 | GILGFVFTL | HLA-A2 |
| EEASI-A | 2 | 0.007380074 | CASSLTSGNEQFF | EEASI-A:E4 | CD4 | PI | CALNTPYSGAGSYQLTF | CASSLTSGNEQFF | clonotype15 | McPas | NA | CASSLTSGNEQFF | Cytomegalovirus (CMV) | D003586 | FRCPRRFCF | HLA-C*07:02 |
| EEASI-C | 7 | 0.008578431 | CEGGSYIPTF | EEASI-C:Bl-5 | CD8 | non | CEGGSYIPTF | CASSQETSGVGGNIQYF | clonotype9 | VDJDB | CEGGSYIPTF | NA | CMV | IE1 | KLGGALQAK | HLA-A*03:01 |
| EEASI-C | 6 | 0.007352941 | CAVAMNSGYSTLTF | EEASI-C:Bl-7 | CD8 | non | CAVAMNSGYSTLTF | CASSEGGTYEQYF | clonotype10 | VDJDB | CAVAMNSGYSTLTF | NA | CMV | IE1 | KLGGALQAK | HLA-A*03:01 |

**Supplementary Table 2.** Re-expressed TCRs matched to database. Yellow indicates the TCR chain in the sequenced data found in the database. Dark orange indicates sequenced TCRs with multiple matching database entries.
