# Supplementary material for: Single-cell RNAseq identifies clonally expanded antigen-specific T-cells following intradermal injection of gold nanoparticles loaded with diabetes autoantigen in humans
Source: Front Immunol. 2023 Oct 16;14:1276255. doi: 10.3389/fimmu.2023.1276255 (PMC10613693; doi:10.3389/fimmu.2023.1276255)
Supplement: Supplementary file 4 [file Table_3.docx]

| EEASI information |  |  |  |  | Database Information |  |  |  |  |  |  |  |
| --- | --- | --- | --- | --- | --- | --- | --- | --- | --- | --- | --- | --- |
| Donor_ID | Clonal expansion | proportion | matched cdr3 | clonotype_id | Source | McPas CDR3.alpha.aa | McPas CDR3.beta.aa | VDJDB alpha/beta | Pathology | Antigen protein | Epitope peptide | MHC |
| EEASI-A | 7 | 0.025830258 | CAVKNYGQNFVF | clonotype3 | VDJDB | NA | NA | TRA | EBV | EBNA4 | AVFDRKSDAK | HLA-A*11:01 |
| EEASI-A | 6 | 0.022140221 | CAVDTGRRALTF | clonotype5 | VDJDB | NA | NA | TRA | HomoSapiens | MLANA | ELAGIGILTV | HLA-A*02:01 |
| EEASI-A | 6 | 0.022140221 | CAVDTGRRALTF | clonotype5 | VDJDB | NA | NA | TRA | CMV | pp65 | NLVPMVATV | HLA-A*02 |
| EEASI-A | 3 | 0.011070111 | CAVTDSNYQLIW | clonotype10 | McPas | CAVTDSNYQLIW | CATSKSNENYEQYF | NA | M. tuberculosis | NA | NA | NA |
| EEASI-A | 3 | 0.011070111 | CAVTDSNYQLIW | clonotype10 | VDJDB | NA | NA | TRA | HomoSapiens | BST2 | LLLGIGILV | HLA-A*02 |
| EEASI-A | 3 | 0.011070111 | CAVTDSNYQLIW | clonotype10 | VDJDB | NA | NA | TRA | CMV | IE1 | KLGGALQAK | HLA-A*03:01 |
| EEASI-A | 2 | 0.007380074 | CASSLTSGNEQFF | clonotype15 | McPas | NA | CASSLTSGNEQFF | NA | Cytomegalovirus (CMV) | D003586 | FRCPRRFCF | HLA-C*07:02 |
| EEASI-A | 2 | 0.007380074 | CASSLTSGNEQFF | clonotype15 | McPas | NA | CASSLTSGNEQFF | NA | Influenza | D009980 | GILGFVFTL | HLA-A2 |
| EEASI-A | 2 | 0.007380074 | CASSLTSGNEQFF | clonotype15 | VDJDB | NA | NA | TRB | InfluenzaA | M | GILGFVFTL | HLA-A*02:01 |
| EEASI-A | 2 | 0.007380074 | CAVVDSNYQLIW | clonotype17 | McPas | CAVVDSNYQLIW | CASSEGGGVDIQYF | NA | M. tuberculosis | NA | NA | NA |
| EEASI-A | 2 | 0.007380074 | CAVVDSNYQLIW | clonotype17 | McPas | CAVVDSNYQLIW | CASSSPWGGTTDTSTDTQYF | NA | Neoantigen | OR5M3-KMV | KMVAVFYTT | HLA-A2 |
| EEASI-A | 2 | 0.007380074 | CAVVDSNYQLIW | clonotype17 | VDJDB | NA | NA | TRA | CMV | IE1 | KLGGALQAK | HLA-A*03:01 |
| EEASI-A | 2 | 0.007380074 | CAVVDSNYQLIW | clonotype17 | VDJDB | NA | NA | TRA | EBV | EBNA4 | AVFDRKSDAK | HLA-A*11:01 |
| EEASI-A | 2 | 0.007380074 | CAVVDSNYQLIW | clonotype17 | VDJDB | NA | NA | TRA | EBV | BZLF1 | RAKFKQLL | HLA-B*08:01 |
| EEASI-A | 2 | 0.007380074 | CASSGTAGTGELFF | clonotype19 | McPas | CAPLDSNYQLIW | CASSGTAGTGELFF | NA | M. tuberculosis | D009169 | NA | NA |
| EEASI-A | 2 | 0.007380074 | CAVKNYGQNFVF | clonotype21 | VDJDB | NA | NA | TRA | EBV | EBNA4 | AVFDRKSDAK | HLA-A*11:01 |
| EEASI-A | 1 | 0.003690037 | CAVGRGFGNVLHC | clonotype101 | VDJDB | NA | NA | TRA | HomoSapiens | BST2 | LLLGIGILV | HLA-A*02 |
| EEASI-A | 1 | 0.003690037 | CAVNNNDMRF | clonotype103 | McPas | CAVNNNDMRF | NA | NA | Melanoma | Melan-A/MART-1 | ELAGIGILTV | HLA-A2 |
| EEASI-A | 1 | 0.003690037 | CAVNNNDMRF | clonotype103 | VDJDB | NA | NA | TRA | InfluenzaA | M | GILGFVFTL | HLA-A*02:01 |
| EEASI-A | 1 | 0.003690037 | CAVNNNDMRF | clonotype103 | VDJDB | NA | NA | TRA | CMV | IE1 | KLGGALQAK | HLA-A*03:01 |
| EEASI-A | 1 | 0.003690037 | CAVKSDYKLSF | clonotype109 | VDJDB | NA | NA | TRA | InfluenzaA | M | GILGFVFTL | HLA-A*02 |
| EEASI-A | 1 | 0.003690037 | CAVDSGGGADGLTF | clonotype118 | McPas | CAVDSGGGADGLTF | CASSWLNTEAFF | NA | Neoantigen | OR14C36-FML_V6L | FMLYLLTLM | HLA-A2 |
| EEASI-A | 1 | 0.003690037 | CAVDSGGGADGLTF | clonotype118 | VDJDB | NA | NA | TRA | HTLV-1 | Tax | LLFGYPVYV | HLA-A*02:01 |
| EEASI-A | 1 | 0.003690037 | CAVDSGGGADGLTF | clonotype118 | VDJDB | NA | NA | TRA | HomoSapiens | BST2 | LLLGIGILV | HLA-A*02 |
| EEASI-A | 1 | 0.003690037 | CAVDSGGGADGLTF | clonotype118 | VDJDB | NA | NA | TRA | HomoSapiens | MLANA | ELAGIGILTV | HLA-A*02:01 |
| EEASI-A | 1 | 0.003690037 | CAVDSGGGADGLTF | clonotype118 | VDJDB | NA | NA | TRA | CMV | IE1 | KLGGALQAK | HLA-A*03:01 |
| EEASI-A | 1 | 0.003690037 | CAAVSGGYNKLIF | clonotype119 | VDJDB | NA | NA | TRA | CMV | IE1 | KLGGALQAK | HLA-A*03:01 |
| EEASI-A | 1 | 0.003690037 | CALDTGTASKLTF | clonotype161 | VDJDB | NA | NA | TRA | CMV | IE1 | KLGGALQAK | HLA-A*03:01 |
| EEASI-A | 1 | 0.003690037 | CAMSAGGAQKLVF | clonotype167 | VDJDB | NA | NA | TRA | CMV | IE1 | KLGGALQAK | HLA-A*03:01 |
| EEASI-A | 1 | 0.003690037 | CASSLAGADTQYF | clonotype168 | VDJDB | NA | NA | TRB | CMV | pp65 | NLVPMVATV | HLA-A*02:01 |
| EEASI-A | 1 | 0.003690037 | CGGSYIPTF | clonotype172 | VDJDB | NA | NA | TRA | InfluenzaA | M | GILGFVFTL | HLA-A*02 |
| EEASI-A | 1 | 0.003690037 | CAVRGSNYQLIW | clonotype174 | VDJDB | NA | NA | TRA | CMV | IE1 | KLGGALQAK | HLA-A*03:01 |
| EEASI-A | 1 | 0.003690037 | CAASSGGYQKVTF | clonotype181 | VDJDB | NA | NA | TRA | CMV | IE1 | KLGGALQAK | HLA-A*03:01 |
| EEASI-A | 1 | 0.003690037 | CAYNAGNMLTF | clonotype27 | VDJDB | NA | NA | TRA | CMV | pp65 | NLVPMVATV | HLA-A*02 |
| EEASI-A | 1 | 0.003690037 | CAYNAGNMLTF | clonotype27 | VDJDB | NA | NA | TRA | HCV | NS3 | KLVALGINAV | HLA-A*02 |
| EEASI-A | 1 | 0.003690037 | CASSFSNQPQHF | clonotype54 | VDJDB | NA | NA | TRB | HomoSapiens | SEC24A | FLYNLLTRV | HLA-A*02:01 |
| EEASI-A | 1 | 0.003690037 | CAVRDSNYQLIW | clonotype55 | McPas | CAVRDSNYQLIW | CASSGTSGGYNEQFF | NA | M. tuberculosis | NA | NA | NA |
| EEASI-A | 1 | 0.003690037 | CAVRDSNYQLIW | clonotype55 | McPas | CAVRDSNYQLIW | CASSKGEVTEAFF | NA | Cytomegalovirus (CMV) | pp65 | NLVPMVATV | HLA-A*02:01 |
| EEASI-A | 1 | 0.003690037 | CAVRDSNYQLIW | clonotype55 | VDJDB | NA | NA | TRA | CMV | IE1 | KLGGALQAK | HLA-A*03:01 |
| EEASI-A | 1 | 0.003690037 | CAVRDSNYQLIW | clonotype55 | VDJDB | NA | NA | TRA | EBV | EBNA3A | RLRAEAQVK | HLA-A*03:01 |
| EEASI-A | 1 | 0.003690037 | CAVRDSNYQLIW | clonotype55 | VDJDB | NA | NA | TRA | EBV | EBNA4 | IVTDFSVIK | HLA-A*11:01 |
| EEASI-A | 1 | 0.003690037 | CAVRDSNYQLIW | clonotype55 | VDJDB | NA | NA | TRA | CMV | pp65 | NLVPMVATV | HLA-A*02:01,HLA-A*02 |
| EEASI-A | 1 | 0.003690037 | CAVRDSNYQLIW | clonotype55 | VDJDB | NA | NA | TRA | EBV | EBNA4 | AVFDRKSDAK | HLA-A*11:01 |
| EEASI-A | 1 | 0.003690037 | CAVRDSNYQLIW | clonotype55 | VDJDB | NA | NA | TRA | HIV-1 | Gag | SLFNTVATLY | HLA-A*02:01 |
| EEASI-A | 1 | 0.003690037 | CAVRDSNYQLIW | clonotype55 | VDJDB | NA | NA | TRA | HomoSapiens | BST2 | LLLGIGILV | HLA-A*02 |
| EEASI-A | 1 | 0.003690037 | CAVRDSNYQLIW | clonotype55 | VDJDB | NA | NA | TRA | StreptomycesKanamyceticus | KanJ | CLLGTYTQDV | HLA-A*02:01 |
| EEASI-A | 1 | 0.003690037 | CAVRDSNYQLIW | clonotype55 | VDJDB | NA | NA | TRA | EBV | BZLF1 | RAKFKQLL | HLA-B*08:01 |
| EEASI-A | 1 | 0.003690037 | CASSFGGSNQPQHF | clonotype59 | VDJDB | NA | NA | TRB | CMV | pp65 | NLVPMVATV | HLA-A*02 |
| EEASI-A | 1 | 0.003690037 | CALSPILTGGGNKLTF | clonotype72 | VDJDB | NA | NA | TRA | InfluenzaA | M | GILGFVFTL | HLA-A*02 |
| EEASI-A | 1 | 0.003690037 | CAVSLTGNQFYF | clonotype73 | VDJDB | NA | NA | TRA | EBV | EBNA4 | AVFDRKSDAK | HLA-A*11:01 |
| EEASI-A | 1 | 0.003690037 | CASSGTAGTGELFF | clonotype75 | McPas | CAPLDSNYQLIW | CASSGTAGTGELFF | NA | M. tuberculosis | D009169 | NA | NA |
| EEASI-A | 1 | 0.003690037 | CAVGPNAGGTSYGKLTF | clonotype84 | VDJDB | NA | NA | TRA | InfluenzaA | M | GILGFVFTL | HLA-A*02 |
| EEASI-A | 1 | 0.003690037 | CAVETSGSRLTF | clonotype91 | VDJDB | NA | NA | TRA | CMV | IE1 | KLGGALQAK | HLA-A*03:01 |
| EEASI-A | 1 | 0.003690037 | CAVDTGRRALTF | clonotype92 | VDJDB | NA | NA | TRA | HomoSapiens | MLANA | ELAGIGILTV | HLA-A*02:01 |
| EEASI-A | 1 | 0.003690037 | CAVDTGRRALTF | clonotype92 | VDJDB | NA | NA | TRA | CMV | pp65 | NLVPMVATV | HLA-A*02 |
| EEASI-B | 1 | 0.016949153 | CAAEGAQKLVF | clonotype37 | VDJDB | NA | NA | TRA | CMV | IE1 | KLGGALQAK | HLA-A*03:01 |
| EEASI-B | 1 | 0.016949153 | CAASSSGAGSYQLTF | clonotype38 | VDJDB | NA | NA | TRA | CMV | pp65 | NLVPMVATV | HLA-A*02 |
| EEASI-C | 7 | 0.008578431 | CEGGSYIPTF | clonotype9 | VDJDB | NA | NA | TRA | CMV | IE1 | KLGGALQAK | HLA-A*03:01 |
| EEASI-C | 6 | 0.007352941 | CAVAMNSGYSTLTF | clonotype10 | VDJDB | NA | NA | TRA | CMV | IE1 | KLGGALQAK | HLA-A*03:01 |
| EEASI-C | 3 | 0.003676471 | CASSLDRGTEAFF | clonotype22 | VDJDB | NA | NA | TRB | CMV | IE1 | KLGGALQAK | HLA-A*03:01 |
| EEASI-C | 3 | 0.003676471 | CAHNAGNNRKLIW | clonotype31 | McPas | CAHNAGNNRKLIW | NA | NA | Human immunodeficiency virus (HIV) | Gag polyprotein RQ13 | RFYKTLRAEQASQ | HLA-DR1 |
| EEASI-C | 3 | 0.003676471 | CAHNAGNNRKLIW | clonotype31 | VDJDB | NA | NA | TRA | HIV-1 | Gag | FRDYVDRFYKTLRAEQASQE | HLA-DRA*01:01 |
| EEASI-C | 3 | 0.003676471 | CALGNTDKLIF | clonotype32 | VDJDB | NA | NA | TRA | HomoSapiens | MLANA | ELAGIGILTV | HLA-A*02:01 |
| EEASI-C | 3 | 0.003676471 | CAGRGNNARLMF | clonotype33 | VDJDB | NA | NA | TRA | InfluenzaA | M | GILGFVFTL | HLA-A*02:01 |
| EEASI-C | 3 | 0.003676471 | CASSLSGSYNEQFF | clonotype33 | VDJDB | NA | NA | TRB | InfluenzaA | M | GILGFVFTL | HLA-A*02 |
| EEASI-C | 2 | 0.00245098 | CVVRDTGFQKLVF | clonotype47 | VDJDB | NA | NA | TRA | InfluenzaA | M | GILGFVFTL | HLA-A*02:01 |
| EEASI-C | 2 | 0.00245098 | CAYITGTASKLTF | clonotype49 | VDJDB | NA | NA | TRA | InfluenzaA | M1 | QARQMVQAMRTIGTHP | HLA-DRA*01 |
| EEASI-C | 2 | 0.00245098 | CASSFAGELFF | clonotype51 | VDJDB | NA | NA | TRB | CMV | IE2 | NEGVKAAW | HLA-B*44:03:08 |
| EEASI-C | 2 | 0.00245098 | CAASSDGQKLLF | clonotype58 | VDJDB | NA | NA | TRA | CMV | IE1 | KLGGALQAK | HLA-A*03:01 |
| EEASI-C | 2 | 0.00245098 | CAGRNNNARLMF | clonotype60 | McPas | CAGRNNNARLMF | CASSLVGSGGEQYF | NA | Cytomegalovirus (CMV) | pp65 | NLVPMVATV | HLA-A2 |
| EEASI-C | 2 | 0.00245098 | CAGRNNNARLMF | clonotype60 | VDJDB | NA | NA | TRA | CMV | IE1 | KLGGALQAK | HLA-A*03:01 |
| EEASI-C | 2 | 0.00245098 | CAGRNNNARLMF | clonotype60 | VDJDB | NA | NA | TRA | CMV | pp65 | NLVPMVATV | HLA-A*02:01 |
| EEASI-C | 1 | 0.00122549 | CAVRDNNARLMF | clonotype119 | VDJDB | NA | NA | TRA | CMV | IE1 | KLGGALQAK | HLA-A*03:01 |
| EEASI-C | 1 | 0.00122549 | CAVRDNNARLMF | clonotype119 | VDJDB | NA | NA | TRA | CMV | pp65 | NLVPMVATV | HLA-A*02 |
| EEASI-C | 1 | 0.00122549 | CASSLGPYEQYF | clonotype124 | McPas | NA | CASSLGPYEQYF | NA | Influenza | D009980 | LPRRSGAAGA | HLA-B7 |
| EEASI-C | 1 | 0.00122549 | CASSLDRGTEAFF | clonotype125 | VDJDB | NA | NA | TRB | CMV | IE1 | KLGGALQAK | HLA-A*03:01 |
| EEASI-C | 1 | 0.00122549 | CAVSDSWGKLQF | clonotype133 | VDJDB | NA | NA | TRA | EBV | EBNA4 | AVFDRKSDAK | HLA-A*11:01 |
| EEASI-C | 1 | 0.00122549 | CAVSDSWGKLQF | clonotype133 | VDJDB | NA | NA | TRA | CMV | IE1 | KLGGALQAK | HLA-A*03:01 |
| EEASI-C | 1 | 0.00122549 | CAVSDSWGKLQF | clonotype133 | VDJDB | NA | NA | TRA | InfluenzaA | M | GILGFVFTL | HLA-A*02:01 |
| EEASI-C | 1 | 0.00122549 | CASSPDGSSYNEQFF | clonotype134 | VDJDB | NA | NA | TRB | InfluenzaA | HA | PKYVKQNTLKLAT | HLA-DRA*01:01 |
| EEASI-C | 1 | 0.00122549 | CALNTGFQKLVF | clonotype146 | VDJDB | NA | NA | TRA | CMV | IE1 | KLGGALQAK | HLA-A*03:01 |
| EEASI-C | 1 | 0.00122549 | CASSLIGGTEAFF | clonotype164 | VDJDB | NA | NA | TRB | CMV | IE1 | KLGGALQAK | HLA-A*03:01 |
| EEASI-C | 1 | 0.00122549 | CAVSSFSGGYNKLIF | clonotype167 | VDJDB | NA | NA | TRA | CMV | IE1 | KLGGALQAK | HLA-A*03:01 |
| EEASI-C | 1 | 0.00122549 | CALSSGYSTLTF | clonotype171 | VDJDB | NA | NA | TRA | EBV | EBNA4 | AVFDRKSDAK | HLA-A*11:01 |
| EEASI-C | 1 | 0.00122549 | CALSSGYSTLTF | clonotype171 | VDJDB | NA | NA | TRA | HomoSapiens | PLA2G6 | FLASKIGRLV | HLA-A*02:01 |
| EEASI-C | 1 | 0.00122549 | CALSSGYSTLTF | clonotype171 | VDJDB | NA | NA | TRA | CMV | IE1 | KLGGALQAK | HLA-A*03:01 |
| EEASI-C | 1 | 0.00122549 | CALSSGYSTLTF | clonotype171 | VDJDB | NA | NA | TRA | InfluenzaA | M | GILGFVFTL | HLA-A*02 |
| EEASI-C | 1 | 0.00122549 | CAVRSGGSNYKLTF | clonotype178 | VDJDB | NA | NA | TRA | InfluenzaA | NP | DPFRLLQNSQVFS | HLA-DRA*01 |
| EEASI-C | 1 | 0.00122549 | CAVRSGGSNYKLTF | clonotype178 | VDJDB | NA | NA | TRA | CMV | IE1 | KLGGALQAK | HLA-A*03:01 |
| EEASI-C | 1 | 0.00122549 | CAVPYNQGGKLIF | clonotype197 | VDJDB | NA | NA | TRA | InfluenzaA | M | GILGFVFTL | HLA-A*02:01 |
| EEASI-C | 1 | 0.00122549 | CAVPYNQGGKLIF | clonotype197 | VDJDB | NA | NA | TRA | EBV | BZLF1 | RAKFKQLL | HLA-B*08:01 |
| EEASI-C | 1 | 0.00122549 | CAVRDGNRDDKIIF | clonotype199 | VDJDB | NA | NA | TRA | EBV | EBNA4 | AVFDRKSDAK | HLA-A*11:01 |
| EEASI-C | 1 | 0.00122549 | CAVRDGNRDDKIIF | clonotype199 | VDJDB | NA | NA | TRA | CMV | IE1 | KLGGALQAK | HLA-A*03:01 |
| EEASI-C | 1 | 0.00122549 | CAVGRGSTLGRLYF | clonotype201 | VDJDB | NA | NA | TRA | CMV | IE1 | KLGGALQAK | HLA-A*03:01 |
| EEASI-C | 1 | 0.00122549 | CAVKAAGNKLTF | clonotype215 | VDJDB | NA | NA | TRA | EBV | EBNA4 | IVTDFSVIK | HLA-A*11:01 |
| EEASI-C | 1 | 0.00122549 | CAVKAAGNKLTF | clonotype215 | VDJDB | NA | NA | TRA | EBV | BZLF1 | RAKFKQLL | HLA-B*08:01 |
| EEASI-C | 1 | 0.00122549 | CAVKAAGNKLTF | clonotype215 | VDJDB | NA | NA | TRA | CMV | IE1 | KLGGALQAK | HLA-A*03:01 |
| EEASI-C | 1 | 0.00122549 | CASRTGNTEAFF | clonotype229 | McPas | NA | CASRTGNTEAFF | NA | Celiac disease | D002446 | NA | NA |
| EEASI-C | 1 | 0.00122549 | CASRGYSNQPQHF | clonotype232 | VDJDB | NA | NA | TRB | CMV | IE1 | KLGGALQAK | HLA-A*03:01 |
| EEASI-C | 1 | 0.00122549 | CALGMDSNYQLIW | clonotype255 | VDJDB | NA | NA | TRA | CMV | IE1 | KLGGALQAK | HLA-A*03:01 |
| EEASI-C | 1 | 0.00122549 | CASSLDRGTEAFF | clonotype279 | VDJDB | NA | NA | TRB | CMV | IE1 | KLGGALQAK | HLA-A*03:01 |
| EEASI-C | 1 | 0.00122549 | CAASSNARLMF | clonotype282 | VDJDB | NA | NA | TRA | CMV | IE1 | KLGGALQAK | HLA-A*03:01 |
| EEASI-C | 1 | 0.00122549 | CAGENSGYALNF | clonotype294 | VDJDB | NA | NA | TRA | CMV | IE1 | KLGGALQAK | HLA-A*03:01 |
| EEASI-C | 1 | 0.00122549 | CASGGGADGLTF | clonotype301 | McPas | CASGGGADGLTF | NA | NA | Melanoma | Melan-A/MART-1 | ELAGIGILTV | HLA-A*02:01 |
| EEASI-C | 1 | 0.00122549 | CASGGGADGLTF | clonotype301 | VDJDB | NA | NA | TRA | HomoSapiens | BST2 | LLLGIGILV | HLA-A*02 |
| EEASI-C | 1 | 0.00122549 | CAVHGSSNTGKLIF | clonotype304 | VDJDB | NA | NA | TRA | CMV | IE1 | KLGGALQAK | HLA-A*03:01 |
| EEASI-C | 1 | 0.00122549 | CASSLIGEAFF | clonotype320 | VDJDB | NA | NA | TRB | EBV | EBNA4 | AVFDRKSDAK | HLA-A*11:01 |
| EEASI-C | 1 | 0.00122549 | CASSLGPYEQYF | clonotype344 | McPas | NA | CASSLGPYEQYF | NA | Influenza | D009980 | LPRRSGAAGA | HLA-B7 |
| EEASI-C | 1 | 0.00122549 | CAARGGSQGNLIF | clonotype345 | VDJDB | NA | NA | TRA | CMV | IE1 | KLGGALQAK | HLA-A*03:01 |
| EEASI-C | 1 | 0.00122549 | CAVFTSGTYKYIF | clonotype351 | VDJDB | NA | NA | TRA | InfluenzaA | HA | PKYVKQNTLKLAT | HLA-DRA*01:01 |
| EEASI-C | 1 | 0.00122549 | CAASANTGNQFYF | clonotype358 | VDJDB | NA | NA | TRA | CMV | IE1 | KLGGALQAK | HLA-A*03:01 |
| EEASI-C | 1 | 0.00122549 | CAVPYNQGGKLIF | clonotype365 | VDJDB | NA | NA | TRA | InfluenzaA | M | GILGFVFTL | HLA-A*02:01 |
| EEASI-C | 1 | 0.00122549 | CAVPYNQGGKLIF | clonotype365 | VDJDB | NA | NA | TRA | EBV | BZLF1 | RAKFKQLL | HLA-B*08:01 |
| EEASI-C | 1 | 0.00122549 | CAASSMEYGNKLVF | clonotype379 | VDJDB | NA | NA | TRA | CMV | IE1 | KLGGALQAK | HLA-A*03:01 |
| EEASI-C | 1 | 0.00122549 | CAVRTGANNLFF | clonotype411 | VDJDB | NA | NA | TRA | CMV | IE1 | KLGGALQAK | HLA-A*03:01 |
| EEASI-C | 1 | 0.00122549 | CAVPYNQGGKLIF | clonotype425 | VDJDB | NA | NA | TRA | InfluenzaA | M | GILGFVFTL | HLA-A*02:01 |
| EEASI-C | 1 | 0.00122549 | CAVPYNQGGKLIF | clonotype425 | VDJDB | NA | NA | TRA | EBV | BZLF1 | RAKFKQLL | HLA-B*08:01 |
| EEASI-C | 1 | 0.00122549 | CALDNYGQNFVF | clonotype430 | VDJDB | NA | NA | TRA | InfluenzaA | M | GILGFVFTL | HLA-A*02 |
| EEASI-C | 1 | 0.00122549 | CALDNYGQNFVF | clonotype430 | VDJDB | NA | NA | TRA | CMV | IE1 | KLGGALQAK | HLA-A*03:01 |
| EEASI-C | 1 | 0.00122549 | CALSTGNQFYF | clonotype457 | McPas | CALSTGNQFYF | CASSAGQGYEQYF | NA | Neoantigen | ATP6AP1-KLG_G3W | KLWASPLHV | HLA-A2 |
| EEASI-C | 1 | 0.00122549 | CAVPYNQGGKLIF | clonotype69 | VDJDB | NA | NA | TRA | InfluenzaA | M | GILGFVFTL | HLA-A*02:01 |
| EEASI-C | 1 | 0.00122549 | CAVPYNQGGKLIF | clonotype69 | VDJDB | NA | NA | TRA | EBV | BZLF1 | RAKFKQLL | HLA-B*08:01 |
| EEASI-C | 1 | 0.00122549 | CAGRNNNARLMF | clonotype78 | McPas | CAGRNNNARLMF | CASSLVGSGGEQYF | NA | Cytomegalovirus (CMV) | pp65 | NLVPMVATV | HLA-A2 |
| EEASI-C | 1 | 0.00122549 | CAGRNNNARLMF | clonotype78 | VDJDB | NA | NA | TRA | CMV | IE1 | KLGGALQAK | HLA-A*03:01 |
| EEASI-C | 1 | 0.00122549 | CAGRNNNARLMF | clonotype78 | VDJDB | NA | NA | TRA | CMV | pp65 | NLVPMVATV | HLA-A*02:01 |
| EEASI-C | 1 | 0.00122549 | CAVYYGGSQGNLIF | clonotype84 | VDJDB | NA | NA | TRA | CMV | IE1 | KLGGALQAK | HLA-A*03:01 |
| EEASI-C | 1 | 0.00122549 | CEGGSYIPTF | clonotype88 | VDJDB | NA | NA | TRA | CMV | IE1 | KLGGALQAK | HLA-A*03:01 |
| EEASI-C | 1 | 0.00122549 | CAPGGSQGNLIF | clonotype92 | VDJDB | NA | NA | TRA | CMV | IE1 | KLGGALQAK | HLA-A*03:01 |
| EEASI-C | 1 | 0.00122549 | CAPGGSQGNLIF | clonotype92 | VDJDB | NA | NA | TRA | InfluenzaA | M | GILGFVFTL | HLA-A*02:01 |
| EEASI-C | 1 | 0.00122549 | CAFAGGTSYGKLTF | clonotype94 | VDJDB | NA | NA | TRA | EBV | BZLF1 | RAKFKQLL | HLA-B*08:01 |
| EEASI-C | 1 | 0.00122549 | CASSLDRGTEAFF | clonotype94 | VDJDB | NA | NA | TRB | CMV | IE1 | KLGGALQAK | HLA-A*03:01 |
| EEASI-C | 1 | 0.00122549 | CASSQGMNTEAFF | clonotype96 | VDJDB | NA | NA | TRB | CMV | IE1 | KLGGALQAK | HLA-A*03:01 |
| EEASI-C | 1 | 0.00122549 | CAVQANYGGATNKLIF | clonotype96 | VDJDB | NA | NA | TRA | CMV | IE1 | KLGGALQAK | HLA-A*03:01 |

**Supplementary Table 3.** Sequenced TCRs matched to databases.
